# The effect of 4 weeks of high‐intensity interval training and 2 weeks of detraining on cardiovascular disease risk factors in male adolescents

**DOI:** 10.1113/EP090340

**Published:** 2023-02-28

**Authors:** Sascha H. Kranen, Ricardo S. Oliveira, Bert Bond, Craig A. Williams, Alan R. Barker

**Affiliations:** ^1^ Children's Health and Exercise Research Centre, Public Health and Sports Sciences University of Exeter Medical School, Faculty of Health and Life Sciences, University of Exeter Exeter UK; ^2^ Department of Physical Education Federal University of Rio Grande do Norte Natal Brazil

**Keywords:** exercise, FMD, HIIT, running, vascular function

## Abstract

High‐intensity interval training (HIIT) represents an effective method to improve cardiometabolic health in adolescents. This study aimed to investigate the effect of 4 weeks of HIIT followed by 2 weeks of detraining on vascular function and traditional cardiovascular disease (CVD) risk factors in adolescent boys. Nineteen male adolescents (13.3 ± 0.5 years) were randomly allocated to either a training (TRAIN, *n* = 10) or control (CON, *n* = 9) group. Participants in TRAIN completed 4 weeks of HIIT running with three sessions per week. Macro‐ (flow‐mediated dilatation, FMD) and microvascular (peak reactive hyperaemia, PRH) function, body composition (fat mass, fat free mass, body fat percentage) and blood biomarkers (glucose, insulin, total cholesterol, high‐ and low‐density lipoprotein, triacylglycerol) were assessed pre‐, 48 h post‐ and 2 weeks post‐training for TRAIN and at equivalent time points for CON. Following training, FMD was significantly greater in TRAIN compared to CON (9.88 ± 2.40% and 8.64 ± 2.70%, respectively; *P* = 0.036) but this difference was lost 2 weeks after training cessation (8.22 ± 2.47% and 8.61 ± 1.99%, respectively; *P* = 0.062). No differences were detected between groups for PRH (*P* = 0.821), body composition (all *P* > 0.14) or blood biomarkers (all *P* > 0.18). In conclusion, 4 weeks of HIIT improved macrovascular function; however, this training period did not measurably change microvascular function, body composition or blood biomarkers. The reversal of the FMD improvement 2 weeks post‐training highlights the importance of the continuation of regular exercise for the primary prevention of CVD.

## Abstract (highlights)

- **What is the central question of this study?**
  What is the effect of 4 weeks of high‐intensity interval training (HIIT) and 2 weeks of detraining on vascular function and traditional cardiovascular disease (CVD) risk factors in male adolescents?
- **What is the main finding and its importance?**
  Four weeks of HIIT improved macrovascular function in adolescents. However, this training period did not measurably change microvascular function, body composition or blood biomarkers. Following 2 weeks of detraining, the improvement in flow‐mediated dilatation (FMD) was lost. This highlights the importance of the continuation of regular exercise for the primary prevention of CVD.

## 1 INTRODUCTION

Cardiovascular diseases (CVD) are the leading cause of non‐communicable deaths worldwide and are expected to remain a global threat (Timmis et al., 2018). The origins of atherosclerosis, as a precursor to overt CVD, can be found in childhood (Celermajer & Ayer, 2006) with atherosclerotic lesions already apparent in the first decades of life (Stary, 1989). The prevalence of these lesions increases with age and their progression is positively related to traditional CVD risk factor status in youth (Berenson et al., 1998). Traditional CVD risk factors in youth include, amongst others, blood lipids like total cholesterol, high‐density lipoprotein (HDL), low‐density lipoprotein (LDL) and triacylglycerol (TAG), glucose tolerance, insulin resistance and body composition (Balagopal et al., 2011). Vascular dysfunction represents an antecedent of atherosclerotic lesions (Juonala et al., 2004; Thijssen et al., 2019) that is already apparent in youth (Celermajer et al., 1992) and can be assessed non‐invasively using the popular and widespread method of flow‐mediated dilatation (FMD) (Celermajer et al., 1992; Thijssen et al., 2019). As the association between macrovascular and microvascular function is weak (Dhindsa et al., 2008), a separate assessment of microvascular function is pertinent. Post‐occlusive reactive hyperaemia utilizing a laser Doppler perfusion monitor is a widely used method to measure microvascular function, with peak reactive hyperaemia (PRH) being the most common outcome measurement (Cracowski et al., 2006), which can be measured reliably alongside the FMD assessment in adolescents (Bond et al., 2017). Identifying interventions that promote vascular function and reduce traditional CVD risk factors is an important prevention strategy in paediatric groups.

Regular exercise training has been shown to reduce CVD events and risk in adults (Jolliffe et al., 2001; Paffenbarger et al., 1986; Sesso et al., 2000). In youth, previous research has demonstrated that as little as 4 min of vigorous physical activity (PA) per day is related to improvements in traditional CVD risk factors (Carson et al., 2014) and that vigorous intensity PA may be more important than moderate intensity PA in terms of modifying CVD risk (Barker et al., 2018; Carson et al., 2014; Hay et al., 2012). A time‐efficient strategy to target vigorous PA is high‐intensity interval training (HIIT). As adolescents have reported higher enjoyment during HIIT compared to moderate‐intensity continuous aerobic exercise (Malik et al., 2017), HIIT may represent a useful approach for the prevention of CVD. A recent systematic review and meta‐analysis reported that school‐based HIIT interventions are an effective means to improve, amongst others, body composition and CVD blood biomarkers in youth (Duncombe et al., 2022). While Logan et al. (2014) suggested that a period of 7+ weeks of HIIT is necessary to improve traditional CVD risk factors in adolescents, it has been shown that 3 weeks of HIIT was sufficient to improve body composition and cardiorespiratory fitness, albeit in obese adolescents (Lazzer et al., 2017). Furthermore, following a 5‐week HIIT intervention, fasting glucose and cardiorespiratory fitness were improved in a group of healthy children (11.1 ± 0.8 years) (van Biljon et al., 2018). Despite the aforementioned studies, few data exist examining the effect of shorter interventions of 2–7 weeks on traditional CVD risk factors.

Dias et al. (2015) concluded in their meta‐analysis that exercise training improves vascular function in overweight and obese youth. However, research on the effect of HIIT interventions on vascular function in healthy adolescents is scarce and contradictory. Bond et al. (2015a) demonstrated that 2 weeks of HIIT improved FMD and autonomic function in healthy adolescents in the absence of traditional CVD risk factors. However, this study lacked a control group and the post‐training assessment was performed ∼24 h after the last training session, potentially measuring the acute effect of the last exercise bout (Rakobowchuk et al., 2008). By contrast, Hopkins et al. (2012) reported no statistically significant differences in FMD following 8 weeks of exercise training in adolescent twins. There is a similar gap in the literature regarding the effect of HIIT on microvascular function in healthy youth. It is currently unknown whether a short HIIT intervention of 4 weeks could enhance both vascular function and traditional CVD risk factors in healthy adolescents.

The health benefits provided by participation in an exercise programme often wane after cessation of the exercise stimulus, for example, the improvements in vascular function after 2 weeks of HIIT in adolescents were mostly lost 3 days post‐training (Bond et al., 2015a). Although detraining periods may appear regularly in youth due to school holidays when sports clubs are paused, little is known about the timeline of the detraining effect in youth following a HIIT intervention and whether differences exist between traditional and novel CVD risk factors.

The aim of this study was to investigate the effect of 4 weeks of HIIT running on macro‐ and microvascular function as well as traditional CVD risk factors in adolescent boys compared to a sedentary control group. A further aim was to identify how 2 weeks of detraining will affect potential benefits from the training period. It was hypothesized that: (1) 4 weeks of HIIT running would improve macro‐ and microvascular function as well as traditional CVD risk factors, and (2) 2 weeks of detraining would reverse the improvements induced by the training back to pre‐training values.

## 2 METHODS

### 2.1 Participants

A convenience sample of 21 adolescent boys (12–14 years) was recruited from a local secondary school to participate in this randomised controlled trial, which was approved by the Sport and Health Sciences Ethics Committee, University of Exeter (161207/B/02) and adhered to the principles of the *Declaration of Helsinki*. The study was not registered in a database. Exclusion criteria included any musculoskeletal injury in the last 6 months and the use of any medication or presence of disease that may influence vascular function. Before commencement of the project, details of the study and associated risks and benefits were explained to pupils and parents and written participant assent and parental consent were obtained.

### 2.2 Experimental design

Participants visited the laboratories at the University of Exeter on three occasions over a period of ∼7 weeks: immediately before the training intervention (PRE), 48 h after the last session of the intervention (POST) and after 2 weeks of detraining (DT). Additionally, participants allocated to the training group completed 12 training sessions over 4 weeks at their school. Participants were asked to replicate their evening meal prior to each laboratory visit. Furthermore, participants were instructed to avoid strenuous exercise during the 48 h preceding each visit. Participants were reminded of these instructions before each laboratory visit.

#### 2.2.1 Visit 1 (PRE): pre‐training and fitness assessment

Participants were transported to the laboratory at 08:00 h after a ∼12 h overnight fast. Stature and body mass of the participants were measured to the nearest 0.1 cm and 0.1 kg, respectively, using standard procedures. Fat mass and fat free mass were estimated using the BodPod (Life Measurement Inc., Concord, CA, USA), which has been validated in this population (Ferri‐Morales et al., 2018). Body mass index (BMI) was calculated as body mass (kg) divided by stature (m) squared. Age and sex specific BMI cut‐points were then used to classify participants as overweight or obese (Cole et al., 2000). Pubertal status was estimated through self‐assessment of secondary sexual characteristics using adapted drawings of the five stages of pubic hair development (Morris & Udry, 1980). Capillary blood samples were taken for the analysis of blood glucose, insulin, total cholesterol, HDL, LDL and TAG. Following a brief familiarisation to the vascular measurements, participants rested in a supine position in a darkened, temperature‐controlled room (24°C) for ∼10 min before the simultaneous assessment of macrovascular (FMD) and microvascular (laser Doppler perfusion monitoring) function (Bond et al., 2017). Subsequently, participants performed a multistage 20 m shuttle run test (Leger et al., 1988) to determine their maximal aerobic speed (MAS), which was used to calculate the intensity of the HIIT sessions. The 20 m shuttle run test is both reliable and valid in adolescents (Castro‐Piñero et al., 2010). Participants were asked to run between two lines set 20 m apart by following the pace of an audio signal. The test began at a speed of 8.5 km h^−1^ and increased by 0.5 km h^−1^ each minute until volitional exhaustion, which was assumed when participants were not able to reach the line in the required time frame on two consecutive occasions. The speed of the last complete shuttle run was taken as MAS. Age and sex specific reference values were then used to describe participants’ fitness levels using the speed at the last complete stage (Tomkinson et al., 2017).

After the first visit, participants were randomly assigned to either a training (TRAIN; *n* = 10) or a control (CON; *n* = 9) group using a closed envelope system. All participants were asked to continue their normal PA routine for the duration of the study and reminded at each visit.

#### 2.2.2 Training intervention

Participants in TRAIN completed 4 weeks of HIIT consisting of three training sessions per week according to the following session breakdown:

Weeks 1 and 2: 8 × 1 min running intervals interspersed with 75 s of passive rest.

Week 3: 10 × 1 min running intervals interspersed with 75 s of passive rest.

Week 4: 12 × 1 min running intervals interspersed with 75 s of passive rest.

Each training session began with a 1 min warm‐up of jogging at 6 km h^−1^. Participants then completed the intervals by running back and forth between two cones following an audio signal. The distance between the cones was determined individually so that the speed necessary to cover it within the time frame equated to 90% of MAS. Heart rate was monitored throughout each training session (Polar Team2, Polar Electro, Kempele, Finland) and participants rated their perceived exertion of the session using a 1−10 Rating of Perceived Exertion (RPE) scale (Foster et al., 2001) immediately following completion of the session. During the rest period participants remained upright near the starting cone.

The training sessions were performed in groups of two to nine participants and took place in the school's sports hall at 08:00 h before the first lesson on Monday, Wednesday and Friday. All sessions were led by a member of the research team.

#### 2.2.3 Visit 2 (POST): post‐training assessment

Visit 2 constituted the post‐training assessment for TRAIN and CON. For TRAIN, it took place 48 h after the last training session to limit the acute effects of this session on vascular function (Rakobowchuk et al., 2008). In order to match the time period from the beginning of the study for TRAIN, participants in CON attended the laboratory 4 weeks and 48 h after completion of visit 1. Participants were transported to the laboratory at 08:00 h after an approximately 12 h overnight fast where body composition, blood and vascular outcomes were reassessed as detailed in visit 1.

#### 2.2.4 Visit 3 (DT): detraining assessment

The final visit took place 2 weeks after the post‐training visit. Participants were transported to the laboratory at 08:00 h after an approximately 12 h overnight fast. Anthropometric measurements, blood sampling and assessment of vascular function were repeated as on visit 2.

### 2.3 Macrovascular function

Macrovascular function was assessed in the brachial artery of the participants’ left arm. High resolution Doppler and B‐mode images of the brachial artery were simultaneously assessed (Sequoia 512, Acuson, Siemens Corp, Aspen, CO, US) with a 13 MHz linear array transducer in duplex mode, in accordance with recent guidelines (Thijssen et al., 2019) and our previous work (Bond et al., 2017). Following a ∼10 min acclimatization period to the temperature‐controlled room (24°C) in the supine position, baseline arterial diameter was measured for 1 min. Endothelium‐dependent vasodilatation of the brachial artery was measured for 3 min after a 5 min ischaemic stimulus induced by rapid forearm pneumatic cuff inflation (moorVMS‐PRES, Moor Instruments Ltd, Axminster, UK) to 200 mmHg. Baseline arterial diameter and post‐occlusion brachial artery diameter were assessed during end diastole using validated ECG‐gating software (Medical Imaging Applications LLC, Coralville, IA, USA) (Mancini et al., 2002; Thijssen et al., 2019). All analyses were performed by the same investigator who was blinded to the group allocation. FMD was calculated using the following equation:

$$\begin{array}{ccc} \mathrm{FMD}\;(\%) & = & \frac{\mathrm{Peak}\;\mathrm{post}-\mathrm{occlusion}\;\mathrm{diameter}-\mathrm{Mean}\;\mathrm{baseline}\;\mathrm{diameter}}{\mathrm{Mean}\;\mathrm{baseline}\;\mathrm{diameter}}\\ & & \times \;100\% \end{array}$$

The area under the curve for estimated shear rate (SR_AUC_) was calculated from the time of cuff deflation until peak dilatation (Pyke & Tschakovsky, 2005). In line with other paediatric data reported by our laboratory (Bond et al., 2015c,b; Kranen et al., 2019) and others (Thijssen et al., 2009a), preliminary analyses using Pearson's correlation coefficient (*r*) revealed that there were no consistently significant correlations between SR_AUC_ and FMD. As a consequence, FMD was not normalised for shear; however, shear data are presented separately in compliance with the current guidelines (Thijssen et al., 2019). Given the suggestion of adjusting FMD allometrically for baseline diameter (Atkinson & Batterham, 2013), Pearson's correlation coefficient (*r*) was applied to examine the relationship between FMD and baseline diameter. However, as there were no consistently significant correlations between FMD and baseline diameter, allometric scaling was not undertaken.

### 2.4 Microvascular function

Microvascular function was simultaneously assessed during the FMD protocol using a laser Doppler perfusion monitor (moorVMS‐LDF, Moor Instruments Ltd, Axminster, UK). An optic probe with eight collecting fibres in a 2 mm ring with a central delivery fibre was attached using adhesive stickers to the distal third of the forearm (Cracowski et al., 2006). High resolution data were collected at 40 Hz and then analysed using the moorVMS‐PC software (moorVMS‐LDF, Moor Instruments Ltd, Axminster, UK). Microvascular function was quantified as PRH, which was defined as the highest point after cuff deflation in relation to the average perfusion pre‐occlusion. The between‐day coefficient of variation for PRH in our laboratory is 19% (Kranen et al., 2021).

### 2.5 Blood analyses

A fingertip capillary blood sample was taken for the analysis of total cholesterol, HDL, LDL and TAG (CardioChek PA, BHR Pharmaceuticals Ltd, Nuneaton, UK) (Panz et al., 2005). Two further fingertip capillary blood samples (∼200 μl each) were taken into heparin/fluoride coated microvettes (CB 300 FH tubes, Sarstedt AG & Co., Nümbrecht, Germany) and analysed immediately for blood glucose (YSI 2900D Biochemistry Analyzer, YSI Inc., Yellow Springs, OH, USA) and centrifuged at 4000 rpm for 8 min, respectively. Plasma was then separated from the centrifuged sample and stored at −80°C for later analysis of plasma [insulin]. Plasma [insulin] was measured in duplicate by enzyme immunoassay (DRG Instruments GmbH, Marburg, Germany) using an EnSpire 2300 plate reader (Perkin Elmer Inc., Waltham, MA, USA) against a five‐parameter standard curve (0, 6.25, 12.5, 25, 50 and 100 μl ml^−1^). The within‐batch coefficient of variation for plasma [insulin] analysis was 5.6%.

### 2.6 Statistical analyses

Sample size was calculated a priori using G*Power based on an observed large effect from the study by Bond et al. (2015a) with FMD as main outcome measure. With the inclusion of statistical power of 80%, an α of 0.05, a partial η^2^ of 0.25 (large effect), two groups (training and control) and three measurements (PRE, POST, DT), a total sample size needed was 18. Recruitment was adjusted to account for an anticipated participant dropout of 10%.

All data are presented as mean and standard deviation (SD) unless otherwise stated. An independent Student's *t*‐test was used to analyse differences in participant characteristics between groups. Descriptive statistics were employed to present the heart rate and perceptual responses during the training sessions.

Due to a technical failure of the pneumatic cuff on visit 1, one participant in CON had no macro‐ and microvascular data at PRE. Furthermore, post‐training blood lipid data were incomplete with missing values from three participants of both TRAIN and CON. A linear mixed model (LMM) analysis with the restricted maximum likelihood method was used to determine the effect of training and detraining on vascular function and other CVD risk factors with the respective pre‐training (PRE) and post‐training (POST) measurement as covariate, respectively. The LMM analysis was chosen because it accommodates missing data by generating estimates using all data available for each participant. For significant group effects, standardised effect sizes (ES) were calculated, with the latter used to determine the magnitude of the observed effect according to the following: trivial (<0.2), small (0.2), moderate (0.5) and large (0.8) (Cohen, 1988). Statistical significance was accepted when *P* < 0.05. IBM SPSS Statistics software (Version 25; IBM Corp., Armonk, NY, USA) was used for all statistical analyses.

## 3 RESULTS

Two participants failed to complete the study due to either illness or time constraints. No significant differences between the characteristics of participants in TRAIN (*n* = 10; age: 13.3 ± 0.6 years; height: 1.59 ± 0.09 m; body mass: 44.4 ± 6.2 kg) and CON (*n* = 9; age: 13.3 ± 0.5 years; height: 1.64 ± 0.10 m; body mass: 50.1 ± 8.8 kg) were observed (all *P* > 0.05). One participant in CON was categorized as overweight. Maturity status for participants in TRAIN and CON was as follows: stage 1, *n* = 1 and 0, stage 2, *n* = 4 and 1, stage 3, *n* = 1 and 4, stage 4, *n* = 3 and 4, respectively. One participant randomised to TRAIN did not report their maturity status. Participants in TRAIN and CON were classified in the following percentiles for cardiorespiratory fitness: 20th, *n* = 0 and 1, 60th, *n* = 1 and 2, 70th, *n* = 3 and 3, 80th, *n* = 3 and 1, 90th, *n* = 3 and 2, respectively. All participants in TRAIN completed 100% of all the training sessions without any adverse events. A summary of HR and RPE responses during the training sessions is provided in Table 1.

**TABLE 1:** Heart rate and perceptual responses during the training sessions.

Training session	Average HR (b · min^−1^)	Average HR (%HR_max_)	Peak HR (b · min^−1^)	Peak HR (%HR_max_)	RPE
1	157 ± 13	77 ± 5	197 ± 9	97 ± 2	3.9 ± 1.0
2	161 ± 12	79 ± 4	199 ± 12	98 ± 3	3.9 ± 0.6
3	163 ± 11	79 ± 4	198 ± 6	97 ± 3	4.2 ± 1.3
4	162 ± 10	79 ± 4	196 ± 9	96 ± 3	3.7 ± 0.8
5	159 ± 7	78 ± 3	196 ± 7	96 ± 3	3.7 ± 0.8
6	158 ± 9	78 ± 4	194 ± 7	95 ± 3	3.5 ± 0.8
7	161 ± 8	79 ± 3	196 ± 7	96 ± 2	3.9 ± 0.7
8	158 ± 9	78 ± 3	193 ± 8	95 ± 3	3.8 ± 0.6
9	155 ± 12	76 ± 4	193 ± 9	95 ± 3	3.8 ± 1.0
10	160 ± 9	78 ± 3	197 ± 10	96 ± 3	3.6 ± 0.8
11	157 ± 11	77 ± 4	193 ± 7	95 ± 2	3.9 ± 0.7
12	159 ± 9	78 ± 3	194 ± 8	95 ± 2	3.6 ± 0.8

Abbreviations: HR, heart rate; b min^−1^, beats per minute; HR_max_, maximum heart rate; RPE, rating of perceived exertion (ranging from 1 (‘very, very easy’) to 10 (‘maximal’)).

### 3.1 Macro‐ and microvascular function

**FIGURE 1:**
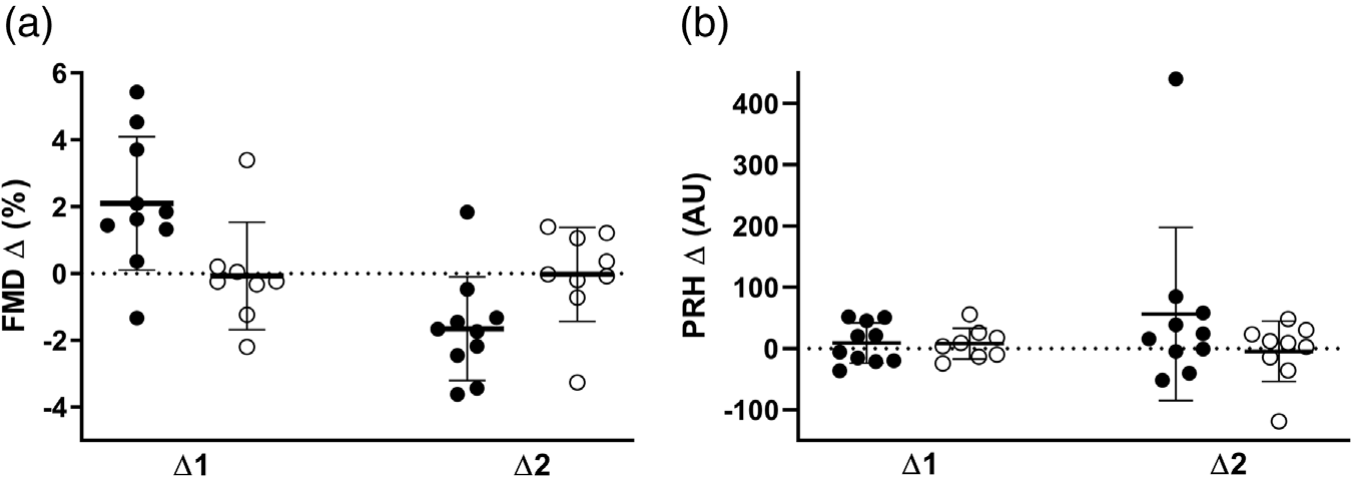
Absolute changes in vascular function from pre‐training to post‐training (∆1) and from post‐training to detraining (∆2) for training group (●) and control group (○). (a) flow‐mediated dilatation (FMD); (b) peak reactive hyperaemia (PRH).

Data for macrovascular function are presented in Table 2. After 4 weeks of HIIT, FMD was significantly higher in TRAIN compared to CON (*F*(15) = 5.33, *P* = 0.036, ES = 0.49) with no significant difference between groups after 2 weeks of detraining (*F*(16) = 4.017, *P* = 0.062). No differences between groups were detected for baseline diameter (*F*(15) = 2.544, *P* = 0.132), peak diameter (*F*(15) = 0.921, *P* = 0.352) or SR_AUC_ (*F*(15) = 0.008, *P* = 0.932). There was no significant effect of training on PRH (*F*(15) = 0.053, *P* = 0.821). Changes in vascular function from pre‐training to post‐training (∆1) and from post‐training to detraining (∆2) are illustrated in Figure 1.

**TABLE 2:** Macrovascular function for training and control group before training (PRE) and after 4 weeks of training (POST) and 2 weeks of detraining (DT).

	TRAIN (*n* = 10)			CON (*n* = 9)		
	PRE	POST	DT	PRE	POST	DT
Baseline diameter (mm)	2.88 ± 0.38	2.91 ± 0.35	2.92 ± 0.32	2.92 ± 0.45 (8)^a^	3.08 ± 0.35	3.06 ± 0.39
Peak artery diameter (mm)	3.09 ± 0.35	3.20 ± 0.35	3.16 ± 0.32	3.17 ± 0.46 (8)^a^	3.34 ± 0.37	3.32 ± 0.41
FMD (%)	7.78 ± 2.36	9.88 ± 2.40*	8.22 ± 2.47	8.54 ± 2.58 (8)^a^	8.64 ± 2.70	8.61 ± 1.99
SR_AUC_	33634 ± 13915	47879 ± 14993	50599 ± 11774	42995 ± 10837 (8)^a^	52876 ± 14485	53300 ± 11799

^a^ Where *n* ≠ denoted number, actual sample number is presented in brackets. *Significant difference between groups, *P* < 0.05. Abbreviations: CON, control group; FMD, flow‐mediated dilatation; SR_AUC_, shear rate area under the curve; TRAIN, training group.

### 3.2 Body composition and blood biomarkers

**FIGURE 2:**
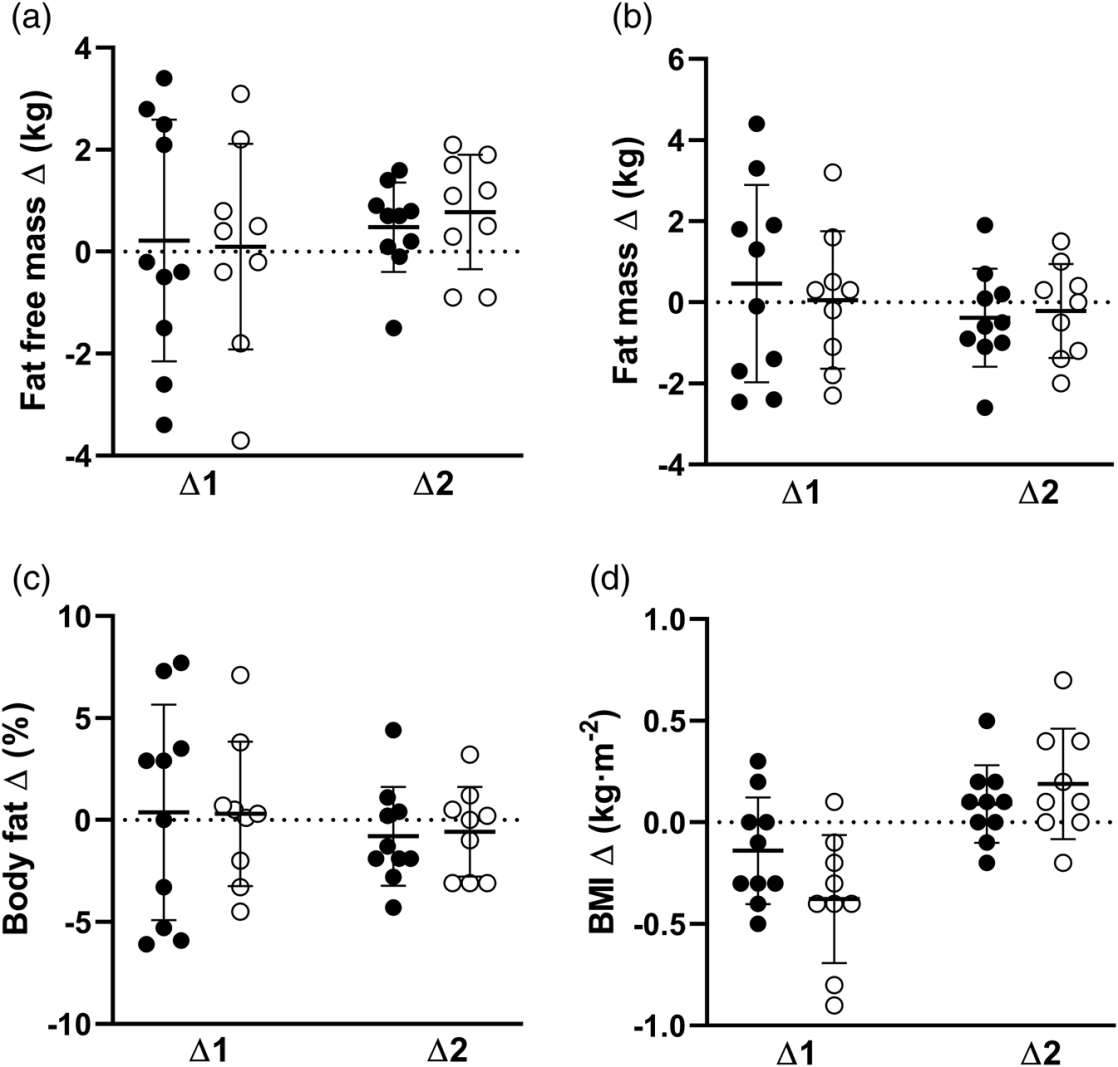
Absolute changes in different body composition parameters from pre‐ to post‐training (∆1) and post‐training to detraining (∆2) for training group (●) and control group (○). (a) Fat free mass, (b) fat mass, (c) body fat percentage, and (d) body mass index.

**FIGURE 3:**
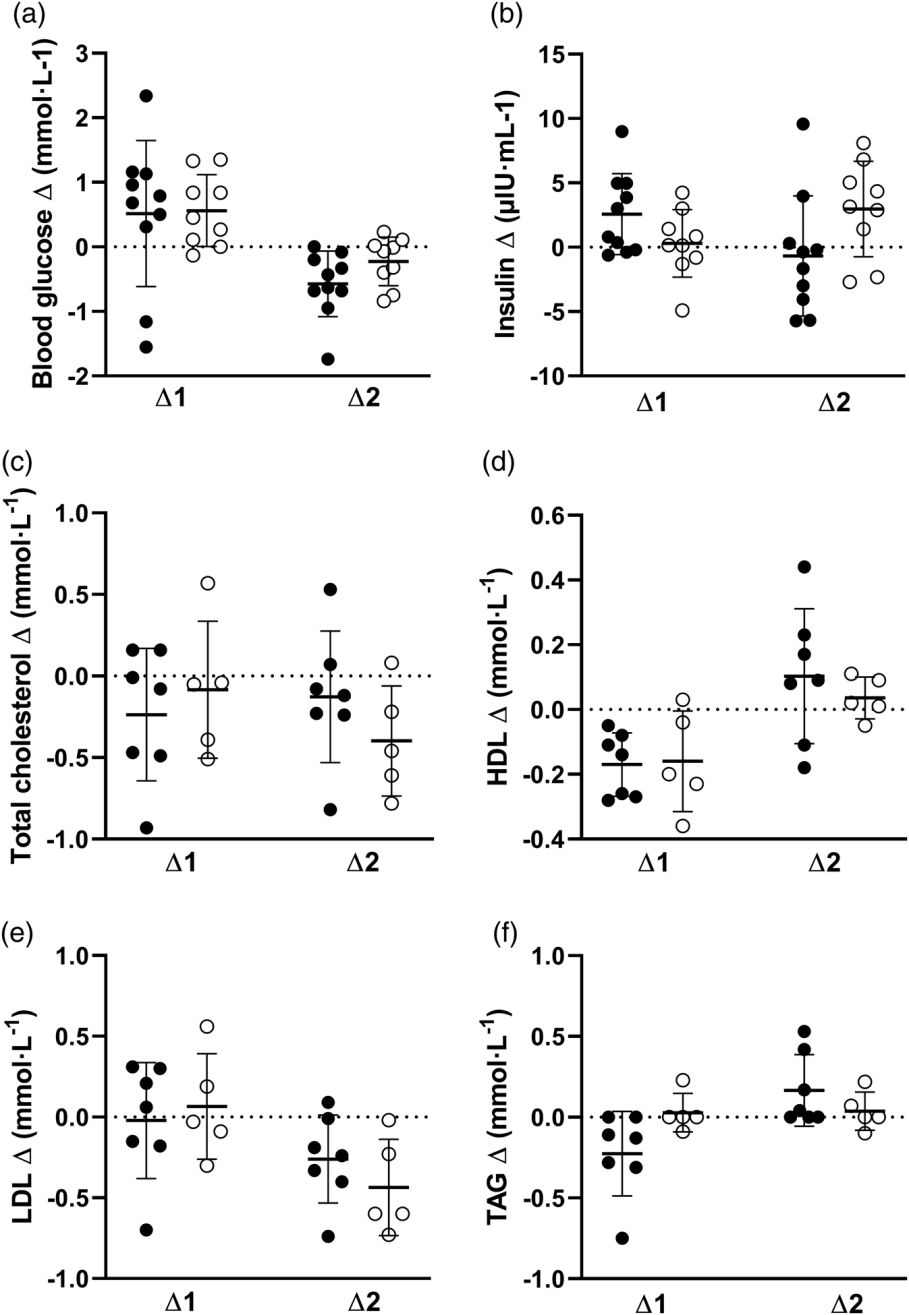
Absolute changes in different blood biomarkers from pre‐ to post‐training (∆1) and post‐training to detraining (∆2) for training group (●) and control group (○). (a) Blood glucose, (b) insulin, (c) total cholesterol, (d) high‐density lipoprotein, (e) low‐density lipoprotein, and (f) triacylglycerol.

Body composition and blood biomarker data are presented in Table 3. No differences between groups were observed after training for any of the body composition outcomes (BMI: *F*(16) = 2.401, *P* = 0.14; fat mass: *F*(16) = 0.023, *P* = 0.88; fat free mass: *F*(16) = 0.001, *P* = 0.98; body fat percentage: *F*(16) = 0.041, *P* = 0.84). Absolute changes from pre‐ to post‐training (∆1) and post‐training to detraining (∆2) are illustrated in Figure 2. There was no difference between TRAIN and CON following the training period (POST) with regards to the blood biomarkers (blood glucose: *F*(16) = 0.41, *P* = 0.531; insulin: *F*(16) = 1.913, *P* = 0.186; total cholesterol: *F*(10) = 0.002, *P* = 0.961; HDL: *F*(10) = 0.393, *P* = 0.545; LDL: *F*(10) = 0.08, *P* = 0.783 and TAG: *F*(10) = 1.82, *P* = 0.207). Absolute changes from pre‐ to post‐training (∆1) and post‐training to detraining (∆2) are illustrated in Figure 2 (body composition parameters) and in Figure 3 (blood biomarkers).

**TABLE 3:** Body composition and blood biomarkers for training and control group before training (PRE) and after 4 weeks of training (POST) and 2 weeks of detraining (DT).

	TRAIN (*n* = 10)			CON (*n* = 9)		
	PRE	POST	DT	PRE	POST	DT
BMI (kg m^−2^)	17.4 ± 0.9	17.2 ± 0.9	17.3 ± 0.8	18.6 ± 2.5	18.2 ± 2.3	18.4 ± 2.4
Fat mass (kg)	7.8 ± 3.1	8.3 ± 3.1	7.9 ± 3.2	11.0 ± 4.7	11.1 ± 5.2	10.8 ± 4.4
Fat free mass (kg)	36.5 ± 7.0	36.7 ± 5.8	37.2 ± 6.0	39.1 ± 6.8	39.2 ± 7.9	40.0 ± 8.3
Body fat (%)	18.0 ± 7.3	18.4 ± 6.2	17.6 ± 6.4	21.6 ± 7.5	21.9 ± 9.1	21.3 ± 7.9
Blood glucose (mmol l^−1^)	5.74 ± 0.32	6.26 ± 1.00	5.69 ± 0.70	5.50 ± 0.45	6.06 ± 0.42	5.84 ± 0.28
Total cholesterol (mmol l^−1^)	3.60 ± 0.55	3.36 ± 0.35 (7)^a^	3.24 ± 0.50	3.42 ± 0.42	3.25 ± 0.58 (6)^a^	3.06 ± 0.53
HDL (mmol l^−1^)	1.35 ± 0.20	1.18 ± 0.20 (7)^a^	1.29 ± 0.34	1.31 ± 0.36	1.09 ± 0.38 (6)^a^	1.16 ± 0.29
LDL (mmol l^−1^)	2.08 ± 0.50	2.06 ± 0.26 (7)^a^	1.80 ± 0.30	1.98 ± 0.58	1.94 ± 0.62 (6)^a^	1.73 ± 0.67
TAG (mmol l^−1^)	0.84 ± 0.31	0.61 ± 0.07 (7)^a^	0.78 ± 0.27	0.65 ± 0.10	1.07 ± 1.02 (6)^a^	0.83 ± 0.34
Insulin (μl ml^−1^)	9.74 ± 3.14	12.32 ± 2.16	11.64 ± 4.55	11.16 ± 3.96	11.47 ± 3.83	14.44 ± 4.44

^a^ Where *n* ≠ denoted number, actual sample number is presented in brackets. Abbreviations: BMI, body mass index; CON, control group; HDL, high‐density lipoprotein; LDL, low‐density lipoprotein; TAG, triacylglycerol; TRAIN, training group.

## 4 DISCUSSION

The main finding of the present study is that 4 weeks of HIIT running improved macrovascular function in a group of male adolescents; however, this improvement was reversed 2 weeks after training cessation. Furthermore, the training had no measurable effect on microvascular function, body composition or blood biomarkers suggesting a longer training duration may be necessary for improvements in these risk factors.

### 4.1 Vascular function and exercise training

In the current study, FMD was significantly enhanced following 4 weeks of HIIT in male adolescents. This finding corroborates the results of Bond et al. (2015a) who showed that 2 weeks of HIIT improved FMD in a group of 13 adolescents. Although the clinical value of change in FMD is unknown for paediatric groups, the observed magnitude of change in FMD of 2.1% after 4 weeks of HIIT is encouraging based on the inverse correlation between FMD and prospective CVD events (Inaba et al., 2010; Ras et al., 2013) and that for every 1% increase in FMD the CVD risk is reduced by an estimated 13% in adults (Green et al., 2011).

Vascular adaptations to exercise training may occur on a functional or structural level (Tinken et al., 2008). Functionally, it is thought that exercise training induces the activation of endothelial nitric oxide synthase (eNOS), which then leads to an improvement in endothelial function (Green et al., 2017). Artery shear stress is the main contributor in the upregulation of eNOS activity (Tinken et al., 2009) and others have shown that shear is positively related to exercise intensity (Thijssen et al., 2009b). We were not able to measure eNOS or shear stress during the exercise bout in the study; however, it can be hypothesized that the intensity used in the current intervention of 90% MAS and the accompanying shear stress may be responsible for the observed improvement of FMD. This notion is reinforced by previous findings that habitual PA at high intensity may be more beneficial to vascular health in children than PA at lower intensities (Hopkins et al., 2009). However, we have recently shown that a single bout of high‐intensity interval running and distance‐matched moderate‐intensity interval running improves FMD irrespective of intensity, indicating that the intermittent nature of the exercise is an important stimulus (Kranen et al., 2021). Others have shown that endothelin‐1, a potent vasoconstrictor (Thijssen et al., 2007), was significantly reduced and plasma levels of NO were significantly increased for up to 4 weeks following a chronic exercise intervention (Maeda et al., 2001). However, as we did not measure endothelin‐1 and NO, this explanation for the increase in FMD remains speculative for the present investigation.

From a structural point of view, regular exercise leads to remodelling of conduit arteries, which increases the vessel diameter and thereby reduces the resistance to flow (Green et al., 2017). However, no significant differences were detected in either baseline or peak brachial artery diameter post‐training in the current study, suggesting the training duration was inadequate to produce measurable structural changes. This interpretation is supported by Tinken et al. (2008) who observed an ongoing increase in conduit dilator capacity, a proxy for artery structure, throughout 8 weeks of exercise training in healthy young males. Previously, Green et al. (2017) stated that both artery function and structure can be altered with an exercise intervention and that the improvement in function acts as a driver for structural changes. Our findings indicate that enhancements in vascular function are achievable with a 4‐week HIIT intervention in a healthy youth population, but measurable structural vascular adaptations are likely to require a longer training stimulus.

The changes in FMD seen after 4 weeks in the present study were not reliably evident when measured 2 weeks after the intervention, which is in line with other studies reporting a reversal of the training‐induced improvements in FMD in patient populations following 8 weeks (Maiorana et al., 2001) or 1 month of detraining (Vona et al., 2004). It could be speculated that the training‐induced expression of eNOS is reversed in the detraining period, thereby causing a reduction in FMD. This idea is supported by the findings of Suvorava et al. (2004) who reported a significant downregulation of eNOS activation concomitant with an impairment in endothelium‐dependent vasodilatation in sedentary compared to active mice. Given the observed detraining effect, it is important from a public health perspective to identify ways to implement HIIT and/or promote habitual vigorous PA.

In comparison to the improvement in macrovascular function, no differences in PRH were detected between groups post‐training. At first glance, this observation should not be surprising because the mechanisms underlying the FMD and PRH responses are divergent: while it has been generally accepted that FMD is mediated by nitric oxide (NO) (Green, 2005), Wong et al. (2003) showed that PRH is not NO‐mediated. Furthermore, adaptations to exercise training are thought to follow different patterns for macro‐ and microvascular function. Exercise leads to an enlargement of the capillarity of the microvasculature in skeletal muscle, which then allows an increased oxygen extraction (Hellsten & Nyberg, 2015). However, the present results differ from the findings of Naylor et al. (2016) who reported an increase in both FMD and microvascular function (cutaneous laser Doppler) after an exercise intervention of 12 weeks in adolescents with type 2 diabetes. Furthermore, Donghui et al. (2019) observed an improvement in microvascular function in obese adolescents after 6 weeks of aerobic exercise training accompanied by a diet restriction. The authors suggested that this enhancement may be related to serum microRNA‐126, an indicator of vascular endothelial function, which was also elevated after the intervention. However, both previous studies dealt with clinical populations (type 2 diabetes, obesity) whereas the participants in our study were healthy adolescents. It has previously been shown that adolescents with type 2 diabetes already exhibit early signs of vascular dysfunction (Naylor et al., 2011). Similarly, the obese participants in the study by Donghui et al. (2019) displayed evidence of microvascular dysfunction before the intervention. Another difference is the shorter training period of only 4 weeks of HIIT compared to 12 weeks of combined aerobic and resistance exercise training with gradual progression (Naylor et al., 2016) and 6 weeks with five sessions per week (Donghui et al., 2019). Akin to our observation, FMD increased significantly after 8 weeks of cycle ergometer training at 80% HR_max_ in healthy young men without changes in the cutaneous microcirculation (Argarini et al., 2021). It was suggested that other stimuli like hypoxia may be required to induce structural microvascular adaptions as it triggers the release of proangiogenic substances during exercise. Furthermore, Morrissey et al. (2018) reported that 3 months of HIIT did not improve microvascular function in obese adolescents who previously did not exhibit microvascular dysfunction. Thus, improvements in microvascular function following exercise training may be reserved to those with impairments. This view is supported by the conclusion of a recent systematic review and meta‐analysis (Lanting et al., 2017) that exercise training would not improve cutaneous microvascular reactivity in physically active healthy adults. Although we have previously shown that a single bout of HIIE can acutely improve microvascular function in healthy adolescents (Bond et al., 2015b,c; Kranen et al., 2021), it remains to be elucidated what exercise training duration is required to chronically enhance microvascular function in healthy youth.

### 4.2 Traditional CVD risk factors and exercise training

Traditional CVD risk factors of body composition and blood biomarkers remained unchanged after 4 weeks of HIIT in the present study. Several paediatric studies have previously reported a reduction in BMI (Corte de Araujo et al., 2012; Lazzer et al., 2017; Racil et al., 2013, 2016) and/or body fat percentage (Lau et al., 2015; Lazzer et al., 2017; Racil et al., 2013; Tjønna et al., 2009) following 3–12 weeks of HIIT. However, as these studies were performed in obese youth populations, the respective findings are apparently not transferable to the healthy adolescents in the current investigation. Moreover, it is noteworthy that the training period in the aforementioned studies with 6–12 weeks, except for the study by Lazzer et al. (2017) with only 3 weeks of training, was longer than in our investigation. In normal‐weight youth, only a few studies observed a reduction in body fat percentage after 7 (Buchan et al., 2011) or 8 weeks (Logan et al., 2016) of HIIT. In accordance with our findings, most studies were unable to detect improvements in body composition (Baquet et al., 2001, 2004; Martin et al., 2015; Weston et al., 2016).

Previously, Bond et al. (2015a) reported that 2 weeks of HIIT had no effect on fasted glucose, insulin or any blood lipids in healthy adolescents. Similarly, the male adolescents in the study by Cockcroft et al. (2019) did not exhibit any improvements in plasma glucose or insulin after a 2‐week HIIT intervention. Although the training period was doubled compared to the aforementioned studies, no enhancements were detected in any of the blood biomarker outcomes examined in the present study. However, in a slightly longer intervention (5 weeks of HIIT with 10 × 1 min intervals at >80% HR_max_, 3 times per week), van Biljon et al. (2018) observed a trend towards improvement in fasting glucose without concomitant enhancement in insulin in healthy 10‐ to 13‐year‐olds. By contrast, Buchan et al. (2011) showed that 7 weeks of HIIT had no effect on glucose, insulin or blood lipids in healthy adolescents and another study reported improvements in TAG after a HIIT intervention of 10 weeks (Weston et al., 2016). For healthy youth, longer training periods seem to be necessary to induce meaningful changes in those blood biomarkers.

It seems that the training duration of 4 weeks was insufficient to improve body composition and blood biomarkers in our healthy paediatric population despite the enhancement in macrovascular function. Given the above findings, however, further research is needed to identify the ideal design and duration of HIIT interventions to improve traditional CVD risk factors. In addition, our findings show that for the evaluation of whether an exercise intervention is deemed successful with regards to reducing CVD risk, it is important to assess a comprehensive range of CVD risk factors, especially including ‘novel’ risk factors like vascular and autonomic function (Joyner & Green, 2009).

### 4.3 Considerations and limitations

This is the first study to examine the effects of 4 weeks of HIIT on vascular function and traditional risk factors in healthy youth. The strengths of the study comprise the additional investigation of a detraining effect, the simultaneous measurement of both macro‐ and microvascular function, the full compliance with the exercise training of all participants, and the timing of the post‐training assessment. However, this study does have limitations. Participants in the current study were adolescent males (12–14 years old) and it is therefore unknown how the results translate to other groups such as female adolescents. Although Bond et al. (2015c) demonstrated that there were no significant differences in vascular function between sexes following an acute bout of exercise, future studies with female adolescents undergoing a chronic exercise intervention are warranted. Similarly, as all participants bar one in the present investigation presented fitness levels of >60th percentile for their age, the findings are restricted to a healthy and fit youth population. Furthermore, post‐training blood lipid data were incomplete with missing values from three participants of both TRAIN and CON, though the LMM analysis was chosen to accommodate missing data. Another limitation of the current study is that we were not able to control for physical activity outside the study, which is also an issue with the wider literature. However, participants were instructed and regularly reminded to continue their habitual exercise routine throughout the study.

### 4.4 Conclusion

Four weeks of HIIT significantly enhanced macrovascular function in a paediatric population without measurable changes in microvascular function, body composition and blood biomarkers. Hence prospective training studies investigating CVD risk should be encouraged to also assess vascular function, otherwise the potential benefit of exercise training on CVD risk might be overlooked. For healthy adolescents without CVD risk factors the training period may be too short to achieve favourable changes in traditional CVD risk factors. The current findings and the extant literature suggest that modifications of these may occur after 6–8 weeks of HIIT. Furthermore, the reversal of the improvement in FMD 2 weeks post‐training highlights the importance of the continuation of regular exercise.

## AUTHOR CONTRIBUTIONS

All the work was done in the Children's Health and Exercise Research Centre, Public Health and Sports Sciences, University of Exeter Medical School, Faculty of Health and Life Sciences, University of Exeter. Sascha H. Kranen, Craig A. Williams and Alan R. Barker designed the experiments; Sascha H. Kranen, Ricardo S. Oliveira and Bert Bond contributed to data collection and analysis; Sascha H. Kranen drafted the manuscript and Alan R. Barker, Ricardo S. Oliveira, Bert Bond and Craig A. Williams critically appraised it. All authors agree to be accountable for all aspects of the work in ensuring that questions related to the accuracy or integrity of any part of the work are appropriately investigated and resolved. All authors approved the final version of the manuscript, all persons designated as authors qualify for authorship, and all those who qualify for authorship are listed.

## CONFLICT OF INTEREST

The authors declare that they have no conflicts of interest.

## Supporting information

Statistical Summary Document

## Data Availability

The data that support the findings of this study are available from the corresponding author upon reasonable request.

## References

[eph13322-bib-0001] Argarini, R. , Carter, H. H. , Smith, K. J. , Naylor, L. H. , Mclaughlin, R. A. , & Green, D. J. (2021). Adaptation to exercise training in conduit arteries and cutaneous microvessels in humans: An optical coherence tomography study. Medicine and Science in Sports and Exercise, 53(9), 1945–1957. 33731650 10.1249/MSS.0000000000002654

[eph13322-bib-0002] Atkinson, G. , & Batterham, A. M. (2013). Allometric scaling of diameter change in the original flow‐mediated dilation protocol. Atherosclerosis, 226(2), 425–427. 23261170 10.1016/j.atherosclerosis.2012.11.027

[eph13322-bib-0003] Balagopal, P. B. , De Ferranti, S. D. , Cook, S. , Daniels, S. R. , Gidding, S. S. , Hayman, L. L. , Mccrindle, B. W. , Mietus‐Snyder, M. L. , & Steinberger, J. , American Heart Association Committee on Atherosclerosis, H., Obesity in Youth of the Council on Cardiovascular Disease in the, Y., Council on Nutrition, P. A., Metabolism, Council on, E. & Prevention . (2011). Nontraditional risk factors and biomarkers for cardiovascular disease: Mechanistic, research, and clinical considerations for youth: A scientific statement from the American Heart Association. Circulation, 123(23), 2749–2769. 21555711 10.1161/CIR.0b013e31821c7c64

[eph13322-bib-0004] Baquet, G. , Berthoin, S. , Gerbeaux, M. , & Van Praagh, E. (2001). High‐intensity aerobic training during a 10 week one‐hour physical education cycle: Effects on physical fitness of adolescents aged 11 to 16. International Journal of Sports Medicine, 22(4), 295–300. 11414674 10.1055/s-2001-14343

[eph13322-bib-0005] Baquet, G. , Guinhouya, C. , Dupont, G. , Nourry, C. , & Berthoin, S. (2004). Effects of a short‐term interval training program on physical fitness in prepubertal children. Journal of Strength and Conditioning Research, 18(4), 708–713. 15574071 10.1519/13813.1

[eph13322-bib-0006] Barker, A. R. , Gracia‐Marco, L. , Ruiz, J. R. , Castillo, M. J. , Aparicio‐Ugarriza, R. , González‐Gross, M. , Kafatos, A. , Androutsos, O. , Polito, A. , Molnar, D. , Widhalm, K. , & Moreno, L. A. (2018). Physical activity, sedentary time, TV viewing, physical fitness and cardiovascular disease risk in adolescents: The HELENA study. International Journal of Cardiology, 254, 303–309. 29221862 10.1016/j.ijcard.2017.11.080

[eph13322-bib-0007] Berenson, G. S. , Srinivasan, S. R. , Bao, W. , Newman, W. P., 3rd , Tracy, R. E. , & Wattigney, W. A. (1998). Association between multiple cardiovascular risk factors and atherosclerosis in children and young adults. The Bogalusa Heart Study. New England Journal of Medicine, 338(23), 1650–1656. 9614255 10.1056/NEJM199806043382302

[eph13322-bib-0008] Bond, B. , Cockcroft, E. J. , Williams, C. A. , Harris, S. , Gates, P. E. , Jackman, S. R. , Armstrong, N. , & Barker, A. R. (2015a). Two weeks of high‐intensity interval training improves novel but not traditional cardiovascular disease risk factors in adolescents. American Journal of Physiology. Heart and Circulatory Physiology, 309(6), H1039–H1047. 26254333 10.1152/ajpheart.00360.2015

[eph13322-bib-0009] Bond, B. , Gates, P. E. , Jackman, S. R. , Corless, L. M. , Williams, C. A. , & Barker, A. R. (2015b). Exercise intensity and the protection from postprandial vascular dysfunction in adolescents. American Journal of Physiology. Heart and Circulatory Physiology, 308(11), H1443–H1450. 25820392 10.1152/ajpheart.00074.2015

[eph13322-bib-0010] Bond, B. , Hind, S. , Williams, C. A. , & Barker, A. R. (2015c). The acute effect of exercise intensity on vascular function in adolescents. Medicine and Science in Sports and Exercise, 47(12), 2628–2635. 26057942 10.1249/MSS.0000000000000715

[eph13322-bib-0011] Bond, B. , Williams, C. A. , & Barker, A. R. (2017). The reliability of a single protocol to determine endothelial, microvascular and autonomic functions in adolescents. Clinical Physiology and Functional Imaging, 37(6), 703–709. 27004991 10.1111/cpf.12362

[eph13322-bib-0012] Buchan, D. S. , Ollis, S. , Young, J. D. , Thomas, N. E. , Cooper, S. M. , Tong, T. K. , Nie, J. , Malina, R. M. , & Baker, J. S. (2011). The effects of time and intensity of exercise on novel and established markers of CVD in adolescent youth. American Journal of Human Biology, 23(4), 517–526. 21465614 10.1002/ajhb.21166

[eph13322-bib-0013] Carson, V. , Rinaldi, R. L. , Torrance, B. , Maximova, K. , Ball, G. D. , Majumdar, S. R. , Plotnikoff, R. C. , Veugelers, P. , Boule, N. G. , Wozny, P. , Mccargar, L. , Downs, S. , Daymont, C. , Lewanczuk, R. , & Mcgavock, J. (2014). Vigorous physical activity and longitudinal associations with cardiometabolic risk factors in youth. International Journal of Obesity, 38(1), 16–21. 23887061 10.1038/ijo.2013.135

[eph13322-bib-0014] Castro‐Piñero, J. , Artero, E. G. , España‐Romero, V. , Ortega, F. B. , Sjöström, M. , Suni, J. , & Ruiz, J. R. (2010). Criterion‐related validity of field‐based fitness tests in youth: A systematic review. British Journal of Sports Medicine, 44(13), 934–943. 19364756 10.1136/bjsm.2009.058321

[eph13322-bib-0015] Celermajer, D. S. , & Ayer, J. G. (2006). Childhood risk factors for adult cardiovascular disease and primary prevention in childhood. Heart, 92(11), 1701–1706. 17041125 10.1136/hrt.2005.081760 PMC1861256

[eph13322-bib-0016] Celermajer, D. S. , Sorensen, K. E. , Gooch, V. M. , Spiegelhalter, D. J. , Miller, O. I. , Sullivan, I. D. , Lloyd, J. K. , & Deanfield, J. E. (1992). Non‐invasive detection of endothelial dysfunction in children and adults at risk of atherosclerosis. Lancet, 340(8828), 1111–1115. 1359209 10.1016/0140-6736(92)93147-f

[eph13322-bib-0017] Cockcroft, E. J. , Bond, B. , Williams, C. A. , Harris, S. , Jackman, S. R. , Armstrong, N. , & Barker, A. R. (2019). The effects of two weeks high‐intensity interval training on fasting glucose, glucose tolerance and insulin resistance in adolescent boys: A pilot study. BMC Sports Science, Medicine and Rehabilitation, 11(1), 29. 10.1186/s13102-019-0141-9 PMC6900855 31827806

[eph13322-bib-0018] Cohen, J. (1988). Statistical power analysis for the behavioural sciences. Lawrence Earlbaum Associates.

[eph13322-bib-0019] Cole, T. J. , Bellizzi, M. C. , Flegal, K. M. , & Dietz, W. H. (2000). Establishing a standard definition for child overweight and obesity worldwide: International survey. BMJ, 320(7244), 1240–1243. 10797032 10.1136/bmj.320.7244.1240 PMC27365

[eph13322-bib-0020] Corte De Araujo, A. C. , Roschel, H. , Picanco, A. R. , Do Prado, D. M. , Villares, S. M. , De Sa Pinto, A. L. , & Gualano, B. (2012). Similar health benefits of endurance and high‐intensity interval training in obese children. PLoS ONE, 7(8), e42747. 22880097 10.1371/journal.pone.0042747 PMC3412799

[eph13322-bib-0021] Cracowski, J. L. , Minson, C. T. , Salvat‐Melis, M. , & Halliwill, J. R. (2006). Methodological issues in the assessment of skin microvascular endothelial function in humans. Trends in Pharmacological Sciences, 27(9), 503–508. 16876881 10.1016/j.tips.2006.07.008

[eph13322-bib-0022] Dhindsa, M. , Sommerlad, S. M. , Devan, A. E. , Barnes, J. N. , Sugawara, J. , Ley, O. , & Tanaka, H. (2008). Interrelationships among noninvasive measures of postischemic macro‐ and microvascular reactivity. Journal of Applied Physiology, 105(2), 427–432. 18483158 10.1152/japplphysiol.90431.2008 PMC2519948

[eph13322-bib-0023] Dias, K. A. , Green, D. J. , Ingul, C. B. , Pavey, T. G. , & Coombes, J. S. (2015). Exercise and vascular function in child obesity: A meta‐analysis. Pediatrics, 136(3), e648–e659. 26260721 10.1542/peds.2015-0616

[eph13322-bib-0024] Donghui, T. , Shuang, B. , Xulong, L. , Meng, Y. , Yujing, G. , Yujie, H. , Juan, L. , & Dongsheng, Y. (2019). Improvement of microvascular endothelial dysfunction induced by exercise and diet is associated with microRNA‐126 in obese adolescents. Microvascular Research, 123, 86–91. 30472037 10.1016/j.mvr.2018.10.009

[eph13322-bib-0025] Duncombe, S. L. , Barker, A. R. , Bond, B. , Earle, R. , Varley‐Campbell, J. , Vlachopoulos, D. , Walker, J. L. , Weston, K. L. , & Stylianou, M. (2022). School‐based high‐intensity interval training programs in children and adolescents: A systematic review and meta‐analysis. PLoS ONE, 17(5), e0266427. 35507539 10.1371/journal.pone.0266427 PMC9067698

[eph13322-bib-0026] Ferri‐Morales, A. , Nascimento‐Ferreira, M. V. , Vlachopoulos, D. , Ubago‐Guisado, E. , Torres‐Costoso, A. , De Moraes, A. C. F. , Barker, A. R. , Moreno, L. A. , Martínez‐Vizcaino, V. , & Gracia‐Marco, L. (2018). Agreement between standard body composition methods to estimate percentage of body fat in young male athletes. Pediatric Exercise Science, 30(3), 402–410. 29543127 10.1123/pes.2017-0171

[eph13322-bib-0027] Foster, C. , Florhaug, J. A. , Franklin, J. , Gottschall, L. , Hrovatin, L. A. , Parker, S. , Doleshal, P. , & Dodge, C. (2001). A new approach to monitoring exercise training. Journal of Strength and Conditioning Research, 15, 109–115. 11708692

[eph13322-bib-0028] Green, D. (2005). Point: Flow‐mediated dilation does reflect nitric oxide‐mediated endothelial function. Journal of Applied Physiology, 99(3), 1233–1234. 16103524 10.1152/japplphysiol.00601.2005

[eph13322-bib-0029] Green, D. J. , Hopman, M. T. , Padilla, J. , Laughlin, M. H. , & Thijssen, D. H. (2017). Vascular adaptation to exercise in humans: Role of hemodynamic stimuli. Physiological Reviews, 97(2), 495–528. 28151424 10.1152/physrev.00014.2016 PMC5539408

[eph13322-bib-0030] Green, D. J. , Jones, H. , Thijssen, D. , Cable, N. T. , & Atkinson, G. (2011). Flow‐mediated dilation and cardiovascular event prediction: Does nitric oxide matter? Hypertension, 57(3), 363–369. 21263128 10.1161/HYPERTENSIONAHA.110.167015

[eph13322-bib-0031] Hay, J. , Maximova, K. , Durksen, A. , Carson, V. , Rinaldi, R. L. , Torrance, B. , Ball, G. D. , Majumdar, S. R. , Plotnikoff, R. C. , Veugelers, P. , Boulé, N. G. , Wozny, P. , Mccargar, L. , Downs, S. , Lewanczuk, R. , & Mcgavock, J. (2012). Physical activity intensity and cardiometabolic risk in youth. Archives of Pediatrics & Adolescent Medicine, 166(11), 1022–1029. 22965682 10.1001/archpediatrics.2012.1028

[eph13322-bib-0032] Hellsten, Y. , & Nyberg, M. (2015). Cardiovascular adaptations to exercise training. Comprehensive Physiology, 6(1), 1–32. 26756625 10.1002/cphy.c140080

[eph13322-bib-0033] Hopkins, N. D. , Stratton, G. , Cable, N. T. , Tinken, T. M. , Graves, L. E. , & Green, D. J. (2012). Impact of exercise training on endothelial function and body composition in young people: A study of mono‐ and di‐zygotic twins. European Journal of Applied Physiology, 112(2), 421–427. 21573774 10.1007/s00421-011-1993-1

[eph13322-bib-0034] Hopkins, N. D. , Stratton, G. , Tinken, T. M. , Mcwhannell, N. , Ridgers, N. D. , Graves, L. E. , George, K. , Cable, N. T. , & Green, D. J. (2009). Relationships between measures of fitness, physical activity, body composition and vascular function in children. Atherosclerosis, 204(1), 244–249. 18930229 10.1016/j.atherosclerosis.2008.09.004

[eph13322-bib-0035] Inaba, Y. , Chen, J. A. , & Bergmann, S. R. (2010). Prediction of future cardiovascular outcomes by flow‐mediated vasodilatation of brachial artery: A meta‐analysis. The International Journal of Cardiovascular Imaging, 26(6), 631–640. 20339920 10.1007/s10554-010-9616-1

[eph13322-bib-0036] Jolliffe, J. A. , Rees, K. , Taylor, R. S. , Thompson, D. , Oldridge, N. , & Ebrahim, S. (2001). Exercise‐based rehabilitation for coronary heart disease. Cochrane Database of Systematic Reviews, 11, Cd001800. 10.1002/14651858.CD001800 11279730

[eph13322-bib-0037] Joyner, M. J. , & Green, D. J. (2009). Exercise protects the cardiovascular system: Effects beyond traditional risk factors. The Journal of Physiology, 587(23), 5551–5558. 19736305 10.1113/jphysiol.2009.179432 PMC2805367

[eph13322-bib-0038] Juonala, M. , Viikari, J. S. , Laitinen, T. , Marniemi, J. , Helenius, H. , Rönnemaa, T. , & Raitakari, O. T. (2004). Interrelations between brachial endothelial function and carotid intima‐media thickness in young adults: The cardiovascular risk in young Finns study. Circulation, 110(18), 2918–23. 15505080 10.1161/01.CIR.0000147540.88559.00

[eph13322-bib-0039] Kranen, S. H. , Bond, B. , Williams, C. A. , & Barker, A. R. (2019). Reliability of low‐flow vasoreactivity in the brachial artery of adolescents. Journal of Clinical Ultrasound, 47(3), 133–138. 30474121 10.1002/jcu.22664

[eph13322-bib-0040] Kranen, S. H. , Oliveira, R. S. , Bond, B. , Williams, C. A. , & Barker, A. R. (2021). The acute effect of high‐ and moderate‐intensity interval exercise on vascular function before and after a glucose challenge in adolescents. Experimental Physiology, 106(4), 913–924. 33369795 10.1113/EP089159

[eph13322-bib-0041] Lanting, S. M. , Johnson, N. A. , Baker, M. K. , Caterson, I. D. , & Chuter, V. H. (2017). The effect of exercise training on cutaneous microvascular reactivity: A systematic review and meta‐analysis. Journal of Science and Medicine in Sport, 20(2), 170–177. 27476375 10.1016/j.jsams.2016.04.002

[eph13322-bib-0042] Lau, P. W. , Wong Del, P. , Ngo, J. K. , Liang, Y. , Kim, C. G. , & Kim, H. S. (2015). Effects of high‐intensity intermittent running exercise in overweight children. European Journal of Sport Science, 15(2), 182–190. 25012183 10.1080/17461391.2014.933880

[eph13322-bib-0043] Lazzer, S. , Tringali, G. , Caccavale, M. , De Micheli, R. , Abbruzzese, L. , & Sartorio, A. (2017). Effects of high‐intensity interval training on physical capacities and substrate oxidation rate in obese adolescents. Journal of Endocrinological Investigation, 40(2), 217–226. 27639403 10.1007/s40618-016-0551-4

[eph13322-bib-0044] Leger, L. A. , Mercier, D. , Gadoury, C. , & Lambert, J. (1988). The multistage 20 metre shuttle run test for aerobic fitness. Journal of Sports Sciences, 6(2), 93–101. 3184250 10.1080/02640418808729800

[eph13322-bib-0045] Logan, G. R. , Harris, N. , Duncan, S. , Plank, L. D. , Merien, F. , & Schofield, G. (2016). Low‐Active male adolescents: A dose response to high‐intensity interval training. Medicine and Science in Sports and Exercise, 48(3), 481–490. 26484952 10.1249/MSS.0000000000000799

[eph13322-bib-0046] Logan, G. R. , Harris, N. , Duncan, S. , & Schofield, G. (2014). A review of adolescent high‐intensity interval training. Sports Medicine, 44(8), 1071–1085. 24743929 10.1007/s40279-014-0187-5

[eph13322-bib-0047] Maeda, S. , Miyauchi, T. , Kakiyama, T. , Sugawara, J. , Iemitsu, M. , Irukayama‐Tomobe, Y. , Murakami, H. , Kumagai, Y. , Kuno, S. , & Matsuda, M. (2001). Effects of exercise training of 8 weeks and detraining on plasma levels of endothelium‐derived factors, endothelin‐1 and nitric oxide, in healthy young humans. Life Sciences, 69(9), 1005–1016. 11508642 10.1016/s0024-3205(01)01192-4

[eph13322-bib-0048] Maiorana, A. , O'driscoll, G. , Cheetham, C. , Dembo, L. , Stanton, K. , Goodman, C. , Taylor, R. , & Green, D. (2001). The effect of combined aerobic and resistance exercise training on vascular function in type 2 diabetes. Journal of the American College of Cardiology, 38(3), 860–866. 11527646 10.1016/s0735-1097(01)01439-5

[eph13322-bib-0049] Malik, A. A. , Williams, C. A. , Bond, B. , Weston, K. L. , & Barker, A. R. (2017). Acute cardiorespiratory, perceptual and enjoyment responses to high‐intensity interval exercise in adolescents. European Journal of Sport Science, 17(10), 1335–1342. 28859545 10.1080/17461391.2017.1364300

[eph13322-bib-0050] Mancini, G. B. , Yeoh, E. , Abbott, D. , & Chan, S. (2002). Validation of an automated method for assessing brachial artery endothelial dysfunction. Canadian Journal of Cardiology, 18(3), 259–262. 11907614

[eph13322-bib-0051] Martin, R. , Buchan, D. S. , Baker, J. S. , Young, J. , Sculthorpe, N. , & Grace, F. M. (2015). Sprint interval training (SIT) is an effective method to maintain cardiorespiratory fitness (CRF) and glucose homeostasis in Scottish adolescents. Biology of Sport, 32(4), 307–313. 26681833 10.5604/20831862.1173644 PMC4672162

[eph13322-bib-0052] Morris, N. M. , & Udry, J. R. (1980). Validation of a self‐administered instrument to assess stage of adolescent development. Journal of Youth and Adolescence, 9(3), 271–280. 24318082 10.1007/BF02088471

[eph13322-bib-0053] Morrissey, C. , Montero, D. , Raverdy, C. , Masson, D. , Amiot, M.‐J. , & Vinet, A. (2018). Effects of exercise intensity on microvascular function in obese adolescents. International Journal of Sports Medicine, 39(6), 450–455. 29710370 10.1055/a-0577-4280

[eph13322-bib-0054] Naylor, L. H. , Davis, E. A. , Kalic, R. J. , Paramalingam, N. , Abraham, M. B. , Jones, T. W. , & Green, D. J. (2016). Exercise training improves vascular function in adolescents with type 2 diabetes. Physiological Reports, 4(4), e12713. 26887327 10.14814/phy2.12713 PMC4759041

[eph13322-bib-0055] Naylor, L. H. , Green, D. J. , Jones, T. W. , Kalic, R. J. , Suriano, K. L. , Shah, M. , Hopkins, N. , & Davis, E. A. (2011). Endothelial function and carotid intima‐medial thickness in adolescents with type 2 diabetes mellitus. Journal of Pediatrics, 159(6), 971–974. 21722916 10.1016/j.jpeds.2011.05.019

[eph13322-bib-0056] Paffenbarger, R. S., JR. , Hyde, R. T. , Wing, A. L. , & Hsieh, C. C. (1986). Physical activity, all‐cause mortality, and longevity of college alumni. New England Journal of Medicine, 314(10), 605–613. 3945246 10.1056/NEJM198603063141003

[eph13322-bib-0057] Panz, V. R. , Raal, F. J. , Paiker, J. , Immelman, R. , & Miles, H. (2005). Performance of the Cardiochek PA and Cholestech LDX point‐of‐care analysers compared to clinical diagnostic laboratory methods for the measurement of lipids. Cardiovascular Journal of South Africa, 16(2), 112–117. 15915279

[eph13322-bib-0058] Pyke, K. E. , & Tschakovsky, M. E. (2005). The relationship between shear stress and flow‐mediated dilatation: Implications for the assessment of endothelial function. The Journal of Physiology, 568(2), 357–369. 16051630 10.1113/jphysiol.2005.089755 PMC1474741

[eph13322-bib-0059] Racil, G. , Ben Ounis, O. , Hammouda, O. , Kallel, A. , Zouhal, H. , Chamari, K. , & Amri, M. (2013). Effects of high vs. moderate exercise intensity during interval training on lipids and adiponectin levels in obese young females. European Journal of Applied Physiology, 113(10), 2531–2540. 23824463 10.1007/s00421-013-2689-5

[eph13322-bib-0060] Racil, G. , Coquart, J. B. , Elmontassar, W. , Haddad, M. , Goebel, R. , Chaouachi, A. , Amri, M. , & Chamari, K. (2016). Greater effects of high‐ compared with moderate‐intensity interval training on cardio‐metabolic variables, blood leptin concentration and ratings of perceived exertion in obese adolescent females. Biology of Sport, 33(2), 145–152. 27274107 10.5604/20831862.1198633 PMC4885625

[eph13322-bib-0061] Rakobowchuk, M. , Tanguay, S. , Burgomaster, K. A. , Howarth, K. R. , Gibala, M. J. , & Macdonald, M. J. (2008). Sprint interval and traditional endurance training induce similar improvements in peripheral arterial stiffness and flow‐mediated dilation in healthy humans. American Journal of Physiology. Regulatory, Integrative and Comparative Physiology, 295(1), R236–R242. 18434437 10.1152/ajpregu.00069.2008 PMC2494806

[eph13322-bib-0062] Ras, R. T. , Streppel, M. T. , Draijer, R. , & Zock, P. L. (2013). Flow‐mediated dilation and cardiovascular risk prediction: A systematic review with meta‐analysis. International Journal of Cardiology, 168(1), 344–351. 23041097 10.1016/j.ijcard.2012.09.047

[eph13322-bib-0063] Sesso, H. D. , Paffenbarger, R. S., Jr. , & Lee, I. M. (2000). Physical activity and coronary heart disease in men: The Harvard alumni health study. Circulation, 102(9), 975–980. 10961960 10.1161/01.cir.102.9.975

[eph13322-bib-0064] Stary, H. C. (1989). Evolution and progression of atherosclerotic lesions in coronary arteries of children and young adults. Arteriosclerosis, *9*(1 suppl), I19–I32. 2912430

[eph13322-bib-0065] Suvorava, T. , Lauer, N. , & Kojda, G. (2004). Physical inactivity causes endothelial dysfunction in healthy young mice. Journal of the American College of Cardiology, 44(6), 1320–1327. 15364339 10.1016/j.jacc.2004.06.030

[eph13322-bib-0066] Thijssen, D. H. , Bullens, L. M. , Van Bemmel, M. M. , Dawson, E. A. , Hopkins, N. , Tinken, T. M. , Black, M. A. , Hopman, M. T. , Cable, N. T. , & Green, D. J. (2009a). Does arterial shear explain the magnitude of flow‐mediated dilation? A comparison between young and older humans. American Journal of Physiology. Heart and Circulatory Physiology, 296(1), H57–H64. 19028795 10.1152/ajpheart.00980.2008 PMC2637778

[eph13322-bib-0067] Thijssen, D. H. , Dawson, E. A. , Black, M. A. , Hopman, M. T. , Cable, N. T. , & Green, D. J. (2009b). Brachial artery blood flow responses to different modalities of lower limb exercise. Medicine and Science in Sports and Exercise, 41(5), 1072–1079. 19346980 10.1249/MSS.0b013e3181923957

[eph13322-bib-0068] Thijssen, D. H. , Rongen, G. A. , Van Dijk, A. , Smits, P. , & Hopman, M. T. (2007). Enhanced endothelin‐1‐mediated leg vascular tone in healthy older subjects. Journal of Applied Physiology, 103(3), 852–857. 17556493 10.1152/japplphysiol.00357.2007

[eph13322-bib-0069] Thijssen, D. H. J. , Bruno, R. M. , Van Mil, A. , Holder, S. M. , Faita, F. , Greyling, A. , Zock, P. L. , Taddei, S. , Deanfield, J. E. , Luscher, T. , Green, D. J. , & Ghiadoni, L. (2019). Expert consensus and evidence‐based recommendations for the assessment of flow‐mediated dilation in humans. European Heart Journal, 40(30), 2534–2547. 31211361 10.1093/eurheartj/ehz350

[eph13322-bib-0070] Timmis, A. , Townsend, N. , Gale, C. , Grobbee, R. , Maniadakis, N. , Flather, M. , Wilkins, E. , Wright, L. , Vos, R. , Bax, J. , Blum, M. , Pinto, F. , & Vardas, P. (2018). European society of cardiology: Cardiovascular disease statistics 2017. European Heart Journal, 39(7), 508–579. 29190377 10.1093/eurheartj/ehx628

[eph13322-bib-0071] Tinken, T. M. , Thijssen, D. H. , Black, M. A. , Cable, N. T. , & Green, D. J. (2008). Time course of change in vasodilator function and capacity in response to exercise training in humans. The Journal of Physiology, 586(20), 5003–5012. 18755749 10.1113/jphysiol.2008.158014 PMC2614057

[eph13322-bib-0072] Tinken, T. M. , Thijssen, D. H. , Hopkins, N. , Black, M. A. , Dawson, E. A. , Minson, C. T. , Newcomer, S. C. , Laughlin, M. H. , Cable, N. T. , & Green, D. J. (2009). Impact of shear rate modulation on vascular function in humans. Hypertension, 54(2), 278–285. 19546374 10.1161/HYPERTENSIONAHA.109.134361 PMC3012006

[eph13322-bib-0073] Tjønna, A. E. , Stølen, T. O. , Bye, A. , Volden, M. , Slørdahl, S. A. , Odegård, R. , Skogvoll, E. , & Wisløff, U. (2009). Aerobic interval training reduces cardiovascular risk factors more than a multitreatment approach in overweight adolescents. Clinical Science, 116(4), 317–326. 18673303 10.1042/CS20080249

[eph13322-bib-0074] Tomkinson, G. R. , Lang, J. J. , Tremblay, M. S. , Dale, M. , Leblanc, A. G. , Belanger, K. , Ortega, F. B. , & Léger, L. (2017). International normative 20 m shuttle run values from 1 142 026 children and youth representing 50 countries. British Journal of Sports Medicine, 51(21), 1545–1554. 27208067 10.1136/bjsports-2016-095987

[eph13322-bib-0075] Van Biljon, A. , Mckune, A. J. , Dubose, K. D. , Kolanisi, U. , & Semple, S. J. (2018). Do short‐term exercise interventions improve cardiometabolic risk factors in children? Journal of Pediatrics, 203, 325–329. 30172428 10.1016/j.jpeds.2018.07.067

[eph13322-bib-0076] Vona, M. , Rossi, A. , Capodaglio, P. , Rizzo, S. , Servi, P. , De Marchi, M. , & Cobelli, F. (2004). Impact of physical training and detraining on endothelium‐dependent vasodilation in patients with recent acute myocardial infarction. American Heart Journal, 147(6), 1039–1046. 15199353 10.1016/j.ahj.2003.12.023

[eph13322-bib-0077] Weston, K. L. , Azevedo, L. B. , Bock, S. , Weston, M. , George, K. P. , & Batterham, A. M. (2016). Effect of novel, school‐based high‐intensity interval training (HIT) on cardiometabolic health in adolescents: Project FFAB (Fun fast activity blasts) ‐ An Exploratory controlled before‐and‐after trial. PLoS ONE, 11(8), e0159116. 27486660 10.1371/journal.pone.0159116 PMC4972319

[eph13322-bib-0078] Wong, B. J. , Wilkins, B. W. , Holowatz, L. A. , & Minson, C. T. (2003). Nitric oxide synthase inhibition does not alter the reactive hyperemic response in the cutaneous circulation. Journal of Applied Physiology, 95(2), 504–510. 12692141 10.1152/japplphysiol.00254.2003

